# Lingering Effects of Prenatal Alcohol Exposure on Basal and Ethanol-Evoked Expression of Inflammatory-Related Genes in the CNS of Adolescent and Adult Rats

**DOI:** 10.3389/fnbeh.2020.00082

**Published:** 2020-06-29

**Authors:** Tamara L. Doremus-Fitzwater, Steven L. Youngentob, Lisa Youngentob, Anny Gano, Andrew S. Vore, Terrence Deak

**Affiliations:** ^1^Department of Psychology, Ithaca College, Ithaca, NY, United States; ^2^Developmental Exposure Alcohol Research Center (DEARC), Binghamton, NY, United States; ^3^University of Tennessee Health Science Center (UTHSC), Memphis, TN, United States; ^4^Department of Psychology, Binghamton University-SUNY, Binghamton, NY, United States

**Keywords:** rat, ethanol, prenatal, neuroimmune, adolescent, cytokine, hippocampus, amygdala

## Abstract

Emerging data suggest that alcohol’s effects on central inflammatory factors are not uniform across the lifespan. In particular, prenatal alcohol exposure (PAE) significantly alters steady-state levels of neuroimmune factors, as well as subsequent reactivity to later immune challenge. Thus, the current experiment investigated developmental sensitivities to, and long-lasting consequences of, PAE on ethanol-evoked cytokine expression in male and female adolescent and adult rats. Pregnant dams received either an *ad libitum* ethanol liquid diet (2.2% GD 6–8; 4.5% GD 9–10; 6.7% GD11–20; 35% daily calories from ethanol) or free-choice access to a control liquid diet and water. At birth, offspring were fostered to dams given free-choice access to the control liquid diet. Pups then matured until mid-adolescence [postnatal day (PD) 35] or adulthood (PD90), at which time they were challenged with either a binge-like dose of ethanol (4 g/kg; intragastrically) or tap water. During intoxication (3 h post-ethanol challenge), brains and blood were collected for assessment of neuroimmune gene expression (reverse transcription-polymerase chain reaction; RT-PCR) in the hippocampus, amygdala, and PVN, as well as for blood ethanol concentrations (BEC) and plasma corticosterone levels. Results revealed that rats challenged with ethanol at either PD35 or PD90 generally exhibited a characteristic cytokine signature of acute intoxication that we have previously reported: increased *Il-6 and* *IkB*α expression, with decreased *Il-1β* and *Tnf*α gene expression. With a few exceptions, this pattern of gene changes was observed in all three structures examined, at both ages of postnatal ethanol challenge, and in both sexes. While few significant effects of PAE were observed for ethanol-induced alterations in cytokine expression, there was a consistent (but nonsignificant) trend for PAE to potentiate the expression of *Il*-6 and *IkB*α in all groups except adult females. Although these data suggest that later-life ethanol challenge was a far greater driver of inflammatory signaling than PAE, the current results demonstrate PAE resulted in subtle long-term alterations in the expression of many key neuroinflammatory factors associated with NF-κB signaling. Such long-lasting impacts of PAE that may engender vulnerability to later environmental events triggering neuroinflammatory processes, such as chronic ethanol exposure or stress, could contribute to heightened vulnerability for PAE-related alterations and deficits.

## Introduction

*In utero* exposure to alcohol produces a multitude of neurobehavioral deficits that exist on a continuum from mild to severe. Although the most severe deficits were first identified as fetal alcohol syndrome (FAS; Jones and Smith, 1973), the effects of prenatal alcohol exposure are now diagnosed as part of a spectrum of possible disorders, termed “fetal alcohol spectrum disorders” (FASD; Mattson et al., 2019; Welch-Carre, 2005). Despite the known risks and consequences of gestational alcohol exposure, a recent report indicated that approximately one in nine women in the US reported drinking at least one alcoholic drink during the past 30 days of their pregnancy, with about one-third of these women reporting binge-level drinking (Denny et al., 2019). Experts believe that, when considering the full range of FASDs, diagnosable rates of FASD are approximately 1% to 5% of the population among school-aged children (CDC, 2019^1^). This comes at a high societal cost, as a 2004 report estimated that the lifetime adjusted cost of caring for a single individual with FAS was approximately $2 million (Lupton et al., 2004).

Many significant neurobehavioral and cognitive effects of FASD have been identified, ranging from intellectual and learning disabilities to attentional deficits and impaired impulse control, and even greater alcohol intake later in life (Baer et al., 2003; Guerri et al., 2009; Mattson et al., 2019). In animal models of pre-and/or post-natal ethanol exposure, results are parallel to those seen in humans (Drew and Kane, 2014). In addition to potentiated ethanol consumption later in ontogeny (Chang et al., 2015; Fabio et al., 2015; Pueta et al., 2008; Youngentob and Glendinning, 2009), rodent offspring exposed to alcohol *in utero* also exhibit increased impulsivity, hyperactivity, attentional impairments, memory deficits, reduced behavioral flexibility, and social deficits (Brys et al., 2014; Juárez and Guerrero-Álvarez, 2015; Idrus et al., 2013; Kelly et al., 2000; Thomas et al., 2010; Waddell and Mooney, 2017). Furthermore, a wide range of structural and functional alterations in the brain have been observed in both human FAS/FASD cases and animal models of prenatal alcohol exposure. For example, a combination of post-mortem and *in vivo* imaging studies has demonstrated many structural abnormalities and functional alterations in the brains of individuals with FASD (Drew and Kane, 2014; Guerri et al., 2009). Animal models have reported parallel brain defects following pre-/early-postnatal alcohol exposure, including reduced cortical, hippocampal, and cerebellar volumes (Kane et al., 2014), as well as abnormalities in the corpus callosum, fiber tracts, and basal ganglia (Drew and Kane, 2014). These studies have demonstrated that the hippocampus and cerebellum are particularly vulnerable to prenatal alcohol exposure (PAE) effects.

Identification of the mechanisms by which PAE negatively influences normative brain development has been the focus of intensive investigation. More recently, ethanol-induced alterations in immune system function from PAE have emerged possible contributors to FASD-related abnormalities in both brain and behavior. A complicating factor in determining the influence of PAE on neuroimmune function across prenatal, adolescent, and adult stages is that the immune system itself is: (a) continually developing, yet not necessarily with a linear accretion of immunocompetence as a function of chronological age (Maggini et al., 2018); (b) sculpting neural circuits and other physiological differences, processes that can be interrupted by developmentally-timed insults (Lenz and Nelson, 2018); and (c) repeatedly responding to changes in the microbiota and external pathogens, contributing to individual differences in steady-state host defense in adulthood (Alpert et al., 2019). Indeed, studies examining the consequences of altering neuroimmune factors have revealed a wide variety of functional consequences (Yirmiya and Goshen, 2011), including abnormalities in neurogenesis, synaptogenesis, synaptic pruning, and myelination (Cai et al., 2000; Deverman and Patterson, 2009; Drew and Kane, 2014; Schwarz and Bilbo, 2012). There is a growing body of research indicating that PAE may cause lasting changes in immune system function and also engender vulnerability to later life immune, stress, or alcohol exposures) Noor and Milligan, 2018). Following PAE, offspring reportedly exhibit changes in steady-state levels of neuroimmune factors throughout ontogeny. For example, adult mice whose mothers drank ethanol during gestation and/or lactation revealed robust increases in toll-like receptor (TLR)-4, TLR-2, NF-kB-p65, and Interleukin (Il)-1β expression levels in the prefrontal cortex (PFC) and hippocampus when compared to offspring from mothers that did not consume ethanol (Cantacorps et al., 2017). Similarly, a more moderate and long-term PAE paradigm revealed lasting elevations in neuroimmune reporters in mice that were PAE offspring, which were observed from the embryonic period to the preweaning period, and then into adulthood (Pascual et al., 2017). Another study examining the effects of PAE in female rats reported significant increases in expression of Il-1β, Il-2, Il-4, Il-5, tumor necrosis factor (TNF)-α and interferon (IFN)-γ in the PFC (Bodnar et al., 2016).

While experiments such as these provide critical evidence that prenatal alcohol significantly alters the neuroimmune milieu in exposed offspring, many of these studies have focused on the effects of PAE very early in life or during adulthood (Drew et al., 2015). Less is known about the effects of PAE on neuroimmune function during the critical developmental period of adolescence. In one study that examined the effects of PAE from gestational day (GD) 7–9, significant upregulation of Il-1β, TNF-α, and transforming growth factor (TGF)-β was observed in the hippocampus and cortex of adolescent PAE offspring (Tiwari and Chopra, 2011). Similarly, a paradigm of moderate gestational alcohol exposure via a liquid diet revealed significant PAE-related increases in TNF-α and Il-1β protein in the hippocampus of PAE offspring at PD30 (Wang et al., 2019). Furthermore, Chang et al. (2015) reported that gestational alcohol exposure increased basal chemokine CCL2 receptor (CCR2) mRNA expression in the lateral hypothalamus, as well as the density of CCR2+ neurons in this same region. Since experiments such as these have demonstrated the sensitivity of the adolescent brain to prenatal/perinatal alcohol exposure, one goal of the current experiments was to investigate whether PAE would alter steady-state levels of neuroimmune gene expression across several different brain regions later life, both during the critical period of adolescence, as well as in adulthood.

Beyond the basal neuroimmune state, other research has demonstrated that PAE affects the offspring’s response to later insult, such as with an immune or alcohol challenge. For example, PAE has been shown to increase susceptibility to infectious disease throughout the lifespan (Gauthier, 2015), with PAE offspring also exhibiting increased vulnerability to chronic neuropathic pain, exacerbated inflammation to adjuvant-induced arthritis, and an altered immune response to an immunogen such as lipopolysaccharide (LPS; Bodnar et al., 2016; Noor and Milligan, 2018; Reid et al., 2019). Alcohol itself has also been shown to be a potent activator of a wide range of cytokines and other inflammation-related genes in adult animals (Crews et al., 2017; Deak et al., 2012; Erickson et al., 2019), with exposure to binge- or suprabinge-like doses of ethanol leading to widespread changes in the expression of neuroinflammatory markers in both the CNS and periphery (Crews et al., 2017). It is not surprising, then, that PAE would also affect the offspring’s response to a later ethanol challenge. For instance, in a recent study, rats were exposed to alcohol from GD10–16 and then given an alcohol challenge in adulthood. While acute ethanol administration resulted in elevations of Il-6 in the cortex, these ethanol-induced increases were exacerbated in adult female PAE rats (Terasaki and Schwarz, 2017).

Importantly, evidence is accumulating to suggest that adolescence is associated with alterations in neuroimmune responsiveness to alcohol challenges that appear different from their adult counterparts. Evidence from our lab and others has shown that adolescent rats and mice (~PD28–PD60) demonstrate reduced neuroimmune function following an acute challenge, regardless of whether the challenge consisted of ethanol, LPS, or stress (Deak et al., in preparation; Doremus-Fitzwater et al., 2015; Kane et al., 2014). In these studies, cytokine reactivity was severely impaired in adolescents, as well as the resultant activation of the hypothalamic-pituitary-adrenal (HPA) axis to the challenge (Girard-Joyal et al., 2015). Therefore, another goal of the current experiments was to examine potential differences in evoked responses of neuroimmune factors to a binge-like ethanol challenge in rats with or without PAE at two different developmental time points—adolescence and adulthood. These studies are especially important because adolescence is a developmental period that is characterized by increased alcohol consumption (Doremus et al., 2005; Vetter et al., 2007), with prenatal alcohol exposure exacerbating this adolescent-typical drinking behavior (Chang et al., 2015; Fabio et al., 2015; Pueta et al., 2008; Youngentob and Glendinning, 2009). PAE has been shown to increase ethanol acceptance by adolescent rats, an effect that seems to reflect a decreased aversion to, and an altered “tuning” of, neural responses to ethanol’s component flavor qualities of bitter and oral irritation, as well as its odor (Glendinning et al., 2012, 2017; Middleton et al., 2009; Youngentob et al., 2007a, 2012). Thus, an examination of the interaction of PAE and adolescent ethanol challenge on neuroimmune responses could be of important functional significance for understanding the mechanisms by which PAE leads to future vulnerability to ethanol effects.

## Materials and Methods

### Subjects

Timed pregnant Long-Evans dams were acquired from Envigo (formerly known as Harlan; Indianapolis, IN, USA) and shipped during the first week of gestation to SUNY Upstate Medical University (an AAALAC-accredited facility). Pregnant dams were housed under standard colony conditions (22°C; 12:00 h light:dark cycle, with lights on 06:00) with food and water available *ad libitum* at all times, except during the period of prenatal alcohol exposure described below. All experimental procedures were approved by the Committee on Humane Use of Animals (CHUA) at SUNY-Upstate Medical University (previous employer for SY), and studies were conducted following the Public Health Service (PHS) policy on the Humane Care and Use of Laboratory Animals.

### Prenatal Alcohol Exposure

The procedures used for prenatal alcohol exposure were employed in previous studies (Middleton et al., 2009; Youngentob et al., 2007a) and are briefly described here. Dams were first assigned to one of two liquid diet conditions that were nutritionally balanced and equivalent to each other concerning their vitamin, mineral, protein, carbohydrate, fat, and fiber content: free-choice liquid (FCL) diet consumption or alcohol-containing liquid diet consumption. To begin, all pregnant dams were first weaned onto the liquid control diet (L10252 recipe; Research Diets, New Brunswick, NJ, USA) from gestational days (GD) 6–10. This diet provided 1.02 kcal/g. For dams in the FCL group, the diet was ethanol-free for the duration of the experiment. For dams producing pups in the PAE condition, however, the concentration of ethanol in the diet was gradually increased during this period (2.2% vol/vol on GD 6–8; 4.5% vol/vol on GD 9–10). From GD11, dams in the FCL group continued to have *ad libitum* access to the liquid diet and water until GD 20. In contrast, ethanol-drinking pregnant dams began receiving *ad libitum* access to a liquid diet containing 35% of daily calories from ethanol (6.7% ethanol vol/vol; total calories = 1.02 kcal/g) through GD 20 (L10251 recipe: Research Diets, New Brunswick, NJ, USA). Previous research using this paradigm of gestational ethanol exposure has demonstrated that dams reach peak blood ethanol concentrations of approximately 150 mg/dl by GD 17 (Youngentob et al., 2007a).

### Offspring

Litters were culled to a total of 10 pups per dam within 24 h of birth, with even numbers of males and females maintained where possible (i.e., no fewer than four, but no more than six, males or females per litter). At culling, pups were cross-fostered to dams fed the FCL diet. This is a standard procedure that ensured the effects of PAE (and other gestational manipulations) were not confounded by differences in maternal care displayed by PAE dams toward their pups. Litters were weaned on PD21, which included rehousing with a same-sex littermate until the time of the later drug challenge. To control for litter effects, no more than one male and one female offspring from each litter were placed into an experimental group.

### Experimental Procedure

**Figure 1:**
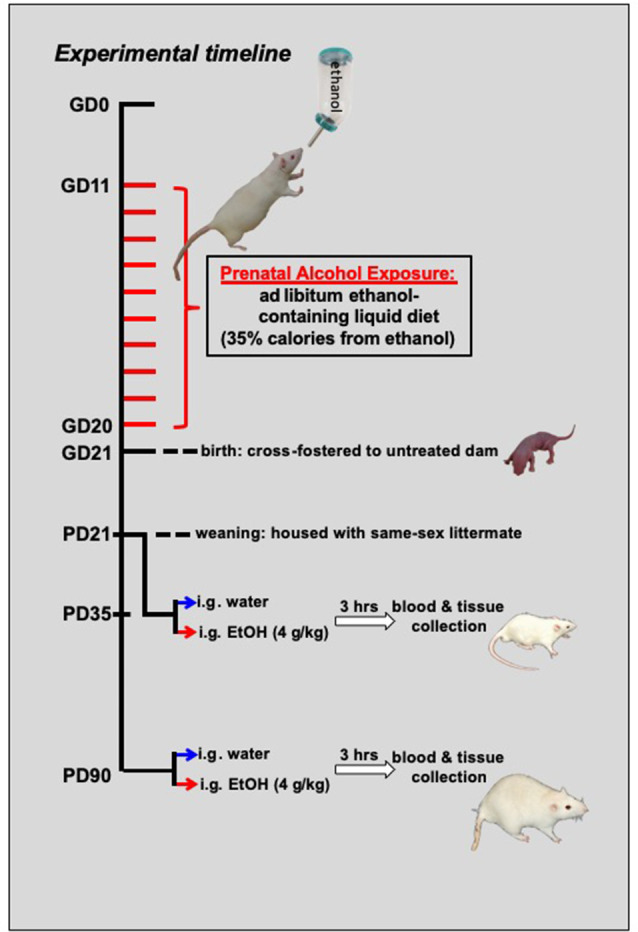
Schematic representing the experimental timeline. From gestational day (GD) 11–GD20, pregnant dams were switched to a liquid diet. For half of the mothers, this diet was a control liquid that did not contain any alcohol, and it was provided *ad libitum*. For the other half of the dams, 35% of the calories in the liquid diet were from the addition of alcohol. At birth, offspring from these dams were cross-fostered to untreated dams and remained with their foster moms until the time of weaning at postnatal day (PD) 21. For the adolescent cohort (*N* = 79; *n* = 9–10 per group), these offspring matured until mid-adolescence, at which time they were acutely challenged with an intragastric (i.g.) gavage of either a binge-like dose of ethanol (4 g/kg) or tap water. Another cohort (*N* = 75; *n* = 8–10 per group) of rats matured into adulthood (P90) before being challenged with ethanol or tap water. For both age groups, trunk blood and brain tissue were collected at peak intoxication, 3 h after the administration of the drug challenge.

Two separate cohorts of offspring were used to examine neuroimmune responsiveness to acute ethanol intoxication at two different developmental periods: mid-adolescence (PD35) or adulthood (PD90). Thus, both cohorts consisted of a 2 (Diet: free-choice control liquid diet vs. ethanol liquid diet) × 2 (Drug Challenge: tap water vs. ethanol) × 2 (Sex: male vs. female) between-subjects factorial design (see Figure 1; for the adolescent cohort, *N* = 79 with *n* = 9–10 per group; for the adult cohort, *N* = 75 with *n* = 8–10). Following prenatal exposure to either the free-choice control or ethanol-containing diet, offspring were left to mature with their littermate until the postnatal ages selected for analysis. At the target ages (i.e., PD35 or PD90), rats were given an acute intragastric (i.g.) intubation of either tap water or ethanol (4 g/kg). Previous work from our laboratory has extensively studied the acute neuroinflammatory response to an acute ethanol challenge. After comparing multiple routes of exposure (i.p. vs. i.g.), multiple doses of ethanol (1–5 g/kg), and multiple time points (e.g., Gano et al., 2019; Doremus-Fitzwater et al., 2014, 2015), we have repeatedly demonstrated that a dose of 4 g/kg delivered i.g. will result in a consistent pattern of alterations in cytokine gene expression across a variety of brain regions. Importantly, these changes occur without the additional inflammatory activation that could potentially be caused by an i.p. injection. Moreover, these neuroinflammatory factors are changing in a way that is distinct from peripheral immune measures, thus indicating that these effects are specific to the brain. This 4 g/kg bolus consisted of ethanol (95%) mixed with tap water to a final concentration of 20% (v/v). Animals that received tap water alone were given their intubation at a volume equivalent to what rats in the ethanol condition received. Previous studies from our laboratory (Doremus-Fitzwater et al., 2014, 2018) have demonstrated that, with this dose and route of exposure, peak intoxication and acute neuroimmune alterations are reached approximately 3 h after ethanol administration. Hence, in both cohorts, samples were collected at this time point following the drug challenge.

### Tissue Collection and Processing

**Figure 2:**
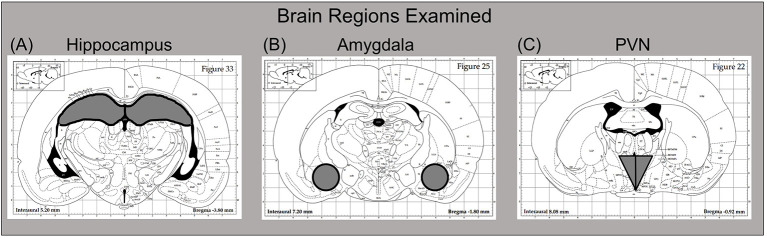
Dissection guide for sites of interest, with images modified from the Paxinos and Watson (1998) rat brain atlas (Published with permission by Elsevier). The **(A)** hippocampus **(B)** amygdala, and **(C)** paraventricular nucleus of the hypothalamus (PVN) were collected for analysis. For the hippocampus and amygdala, the right side of the structure was used for these reverse transcription-polymerase chain reaction (RT-PCR) assays, with the left side harvested for other purposes. In the case of the PVN, the entire punch that was collected was used here for RT-PCR.

Three hours after drug challenge, rats were euthanized by brief CO_2_ exposure. Following decapitation, trunk blood was collected in K3-EDTA containing glass blood collection tubes (BD Vacutainers, VWR cat. no. VT6450, Radnor, PA, USA), with plasma then separated in a refrigerated centrifuge and frozen at −20°C until time of assay. Whole brains were immediately flash frozen on dry ice and stored at −80°C. Brain structures of interest [e.g., hippocampus, amygdala, and paraventricular nucleus of the hypothalamus (PVN)] were collected by microdissection. These regions were chosen for analysis because of numerous prior studies reporting sensitivity of these areas to PAE neuroimmune effects (Bodnar et al., 2016; Cantacorps et al., 2017; Drew et al., 2015; Terasaki and Schwarz, 2016), as well as their responsiveness to acute ethanol challenge on neuroimmune functioning (Doremus-Fitzwater et al., 2014, 2015, 2018; Gano et al., 2017; Kane et al., 2014; Terasaki and Schwarz, 2017). To do this, frozen brains were sliced in a cryostat (maintained at −20°C), with brain regions collected using chilled micropunches (1.0–2.0 mm) according to the Paxinos and Watson (1998) rat brain atlas (see Figure 2). Brain punches were collected from the right side of each structure and were stored at −80°C until the time of RNA extraction.

### Reverse-Transcription Polymerase Chain Reaction

A Qiagen TissueLyser (Qiagen, Valencia, CA, USA) provided rapid, thorough, and consistent homogenization of brain samples. Each structure was placed into a 2.0 ml Eppendorf tube containing 500 μl of Trizol^®^ RNA reagent (Invitrogen, Grand Island, NY, USA) and a 5 mm stainless steel bead, and was then rapidly shaken for 2 min for complete disruption/homogenization of the tissue. Chloroform (100 μl) was then added to the Trizol solution, the samples briefly were shaken, and then samples were centrifuged for 15 min at 4°C. An equal volume of 70% ethanol was added to the supernatant and purified through RNeasy mini columns (Qiagen, Valencia, CA, USA) according to the manufacturer’s instructions. Columns were washed with buffer and eluted with 30 μl of RNase-free water (65°C). RNA yield and quality were determined using a Nanodrop micro-volume spectrophotometer (NanoDrop 2000, Thermo Fisher Scientific, Wilmington, DE, USA), with total RNA stored at −80°C until the time of cDNA synthesis. Synthesis of cDNA was performed on 0.1–1.0 μg of normalized total RNA from each sample using the QuantiTect^®^ Reverse Transcription Kit (Cat. No. 205313, Qiagen, Valencia, CA, USA) which included a DNase treatment step. All cDNA was stored at −20°C until the time of assay.

Probed cDNA amplification was performed in a 10 μl reaction consisting of 5 μl IQ SYBR Green Supermix (Bio-Rad, cat. no. 170-8882, Hercules, CA, USA), 0.5 μl primer (final concentration 250 nM), 0.5 μl cDNA template, and 4 μl Rnase-free water run in triplicate in a 384 well plate (BioRad, cat. no. HSP-3805), and captured in real-time using a PCR detection system (BioRad, model no. CFX384). Following a 3-min hot start (95°C), samples underwent denaturation for 30 s at 95°C, annealing for 30 s at 60°C and extension for 30 s at 72°C for 50 cycles. An additional denaturation (95°C, 1 min) and annealing cycle (55°C, 1 min) were conducted to ensure proper product alignment before melt curve analysis. For melt curve analysis, samples underwent 0.5°C changes every 15 s ranging from 55°C to 95°C. A single peak expressed as the negative first derivative of the change in fluorescence as a function of temperature indicated primer specificity to the target gene. Glyceraldehyde 3-phosphate dehydrogenase (*Gadph*) was used as a reference gene in these experiments, as studies from our laboratory have revealed more stable gene expression across ethanol treatment conditions with this gene (Doremus-Fitzwater et al., 2014, 2015; Gano et al., 2017). Before conducting analyses of cytokine data, *Gapdh* expression was first examined as a separate target to confirm no statistical differences in expression of this reference gene across conditions. Thereafter, gene expression of neuroinflammatory targets was quantified relative to the expression of *Gapdh* using the 2^−ΔΔC(t)^ method (Livak and Schmittgen, 2001), with male, FCL, vehicle-challenged controls serving as the ultimate control group. Specifically, the equation used was: 2^−ΔΔC(t)^ target gene–C(t) *Gapdh* for individual) − (mean of ultimate control group: C(t) target gene–C(t) *Gapdh*)] × 100. Thus, in all figures and tables showing inflammatory genes, this equation was used to calculate relative gene expression. A list of genes examined and their primer sequences can be found in Table 1.

**Table 1:** Reverse transcription-polymerase chain reaction (RT-PCR) primers and sequences for genes examined in brain.

Gene target	Accession #	RT-PCR Primer sequences
*Il-6*^a^	NM_012589.2	Forward: 5′-TAGTCCTTCCTACCCCAACTTCC-3′
		Reverse: 5′-TTGGTCCTTAGCCACTCCTTC-3′
*IκBα*^b^	NM_001105720.2	Forward: 5′-CTGTTGAAGTGTGGGGCTGA-3′
		Reverse: 5′-AGGGCAACTCATCTTCCGTG-3′
*Il-1β*^c^	NM_031512.2	Forward: 5′-TCCTCTGTGACTCGTGGGAT-3′
		Reverse: 5′-TGGAGAATACCACTTGTTGGCT-3′
*Tnfα*;^d^	NM_012675.3	Forward: 5′-GTCCCAACAAGGAGGAGAAGTT-3′
		Reverse: 5′-CTCCGCTTGGTGGTTTGCTA-3′
*CX_3_CL-1*^e^	NM_134455.1	Forward: 5′-GCCATCATCCTGGAGACGAG-3′
		Reverse: 5′-CGCTTCTCAAACTTGCCACC-3′
*CX_3_CL-1R*^f^	NM_133534.1	Forward: 5′-TCTTCCTCTTCTGGACGCCT-3′
		Reverse: 5′-TAAACGCCACTGTCTCCGTC­3′
*Gapdh*^g^	NM_017008	Forward: 5′-GTGCCAGCCTCGTCTCATAG-3′
		Reverse: 5′-AGAGAAGGCAGCCCTGGTAA-3′

*^a^Interleukin-6. ^b^Nuclear factor of kappa light polypeptide gene enhancer in B-cells inhibitor, alpha. ^c^Interleukin-1 beta. ^d^Tumor necrosis factor-alpha. ^e^Chemokine (C-X3-C motif) ligand 1 (also known as fractalkine). ^f^Chemokine (C-X3-C motif) ligand 1 receptor (also known as fractalkine receptor). ^g^Glyceraldehyde 3-phosphate dehydrogenase*.

### Plasma Measurement of Blood Ethanol Concentrations and Corticosterone

All blood ethanol concentrations (BECs) were determined in 5 μl aliquots of plasma using an Analox AM-1 alcohol analyzer (Analox Instruments, Lunenburg, MA, USA). The machine was first calibrated using a 100 mg% industry-standard, with BECs recorded in milligrams per deciliter (mg%). Accuracy was confirmed with a quality control solution provided by Analox Instruments, which contained a known concentration of ethanol. After confirmation with the quality control, experimental samples were measured and counterbalanced across groups concerning the order in which they were processed. Accuracy of the machine was systematically rechecked by reading the quality control following the measurement of every 12–15 samples, as well as after the final sample.

Quantitative determination of plasma CORT was assessed by a commercially available ELISA kit (Cat No: ADI-901-097; Enzo Life Sciences, Farmingdale, NY, USA). The CORT assay had a sensitivity of 27.0 pg/ml and an inter-assay coefficient of 10.29%. The samples were diluted 1:30 and heat-inactivated to denature endogenous corticosteroid-binding globulin (CBG) by immersion in 75°C water for 60 min, which produces a much more reliable and uniform denaturation of CBG than the enzyme cleavage step provided by the kit (unpublished observations). After heat inactivation of CBG, samples were processed according to the directions provided by the kit.

### Data Analysis

Because rats challenged at PD35 vs. PD90 were obtained from two separate cohorts, data from these two age groups were analyzed separately. Thus, while general patterns of ethanol and PAE effects can be compared across ages, these data sets did not allow for direct statistical comparison between adolescents and adults.

For all variables of interest, data were first checked for outliers using the extreme studentized deviate (ESD) method (Grubb’s test), with values outside the boundaries of more or less than two standard deviations from the group mean meeting the criterion for outliers. Given the logarithmic amplification of RT-PCR data, it is not usual that there are instances in which a sample was an extreme data point in the analysis of only one target within a structure. In these situations, the data point was only removed in the analysis of that particular dependent variable. However, if a sample was an outlier for more than two individual gene targets, it was then dropped for analysis of all targets in that structure. Analyses of the adolescent data included four outliers or missing data points: one BEC sample from a male PAE ethanol-challenged rat was lost during processing; one female FCL ethanol-challenged rat was an outlier for all targets in the hippocampus; one male FCL ethanol-challenged animal was an outlier for all gene targets in the amygdala, and one female PAE water-challenged rat was not included in the analyses of all targets in the PVN. For analyses of adult data, gene targets in the PVN only revealed one outlier—a male PAE vehicle-challenged rat was eliminated from the analysis of *IκBα* in this tissue compartment. For the adult amygdala data, three rats were eliminated from analyses of all amygdala gene targets: one female PAE rat challenged with water, one male PAE rat challenged with ethanol, and one female FCL animal challenged with ethanol. Additionally, in the analysis of *IκBα* in the amygdala, a female FCL vehicle-exposed rat was excluded. During processing the of hippocampal tissue one sample from the female FCL ethanol-challenged group was lost. Analyses of outliers for gene targets in the hippocampus involved the exclusion of several outliers for all genes: one male and one female from the FCL vehicle-challenged groups, as well as one male from the PAE water-exposed condition. For *Il-1β, Tnfα,* and *Il-6* analyses, an additional male FCL water-exposed rat was also excluded, whereas an additional male PAE water-exposed and female FCL water-exposed rat were also excluded for *Tnfα,* and *Il-6* analyses, respectively. In the analyses of cytokine targets, plasma corticosterone concentrations, and plasma ethanol concentrations, data were analyzed (Statistica, TIBCO^®^) using a 2 (Prenatal Diet: FCL vs. PAE) × 2 (Sex: Male vs. Female) × 2 (Drug Challenge: Veh vs. EtOH) factorial ANOVA (*p* < 0.05), with Fisher’s Least Significant Difference (LSD) test used for *post hoc* examination of any significant 2- or 3-way interactions (*p* < 0.05) that were observed.

## Results

For both adolescent and adult rats, results from the analyses of all gene targets that were examined across three different brain regions are in Table 2. A description of these significant outcomes follows below.

**Table 2:** Group means and SEMs for neuroimmune targets in adolescent and adult rats following a water or ethanol challenge.

	**Adolescents**				**Adults**			
	**FCL**		**PAE**		**FCL**		**PAE**	
	**VEH**	**EtOH**	**VEH**	**EtOH**	**VEH**	**EtOH**	**VEH**	**EtOH**
**Hippocampus**
*Il-1β^a^*	96.7 (8.6)	**68.8 (6.7)**	94.3 (13.8)	**60.7 (8.0)**	109.3 (8.2)	**69.9 (8.7)**	102.9 (9.3)	**53.3 (5.6)**
*Tnfα^b^*	101.4 (8.9)	**61.3 (11.1)**	92.2 (8.8)	**47.8 (6.2)**	M: 103.9 (11.6)	**M: 34.9 (5.9)**	*M: 112.0 (18.0)*	**M: 71.9 (11.6)**
					F: 166.7 (17.1)	**F: 59.0 (8.7)**	*F: 101.4 (13.0)*	**F: 58.5 (8.70)**
*CX_3_CL-1^c^*	105.2 (4.4)	105.1 (3.3)	104.4 (5.0)	102.0 (3.2)	M: 91.1 (7.6)	**M: 79.9 (8.6)**	M: 99.6 (6.1)	**M: 86.0 (5.3)**
					F: 104.0 (6.8)	**F: 96.6 (8.2)**	F: 93.3 (5.7)	**F: 75.9 (8.0)**
*CX_3_CL-1R^d^*	102.4 (3.4)	**95.4 (2.8)**	*99.9 (4.6)*	**85.1 (2.3)**	M: 104.7 (9.9)	**M: 81.5 (10.5)**	M: 115.7 (6.5)	**M: 88.5 (5.9)**
					F: 120.3 (8.7)	**F: 95.2 (8.2)**	F: 102.2 (5.9)	**F: 81.3 (10.3)**
**Amygdala**	
*Il-1β*	112.3 (12.5)	**75.1 (5.8)**	108.7 (11.5)	**64.2 (5.0)**	116.6 (10.0)	**87.5 (10.4)**	111.0 (10.3)	**76.2 (7.5)**
*Tnfα*	109.7 (8.9)	**65.4 (4.5)**	107.9 (8.6)	**51.7 (5.4)**	101.1 (7.2)	**35.0 (3.8)**	106.8 (7.9)	**49.1 (4.3)**
*CX_3_CL-1*	104.5 (3.4)	96.9 (3.8)	99.7 (4.3)	104.7 (6.1)	M: 102.0 (7.2)	M: 112.5 (4.4)	M: 102.6 (8.6)	M: 98.8 (7.3)
					F: 145.3 (19.6)	F: 122.3 (10.1)	F: 143.5 (12.8)	F: 106.0 (9.4)
*CX_3_CL-1R*	106.8 (3.5)	100.9 (3.4)	116.4 (4.5)	105.0 (6.3)	109.0 (5.8)	**91.2 (4.4)**	116.8 (7.1)	**98.9 (5.3)**
**PVN**	
*Il-1β*	M: 102.0 (6.7)	**M: 64.1 (6.0)**	M: 101.0 (10.6)	**M: 79.1 (6.5)**	107.8 (10.5)	**88.7 (9.4)**	*95.9 (7.3)*	**66.0 (5.8)**
	F: 100.0 (7.1)	**F: 75.2 (7.2)**	F: 79.1 (7.2)	**F: 65.6 (7.2)**	107.8 (10.5)	**88.7 (9.4)**	*95.9 (7.3)*	**66.0 (5.8)**
*Tnfα*	M: 102.1 (6.5)	**M: 62.0 (4.4)**	M: 92.8 (8.1)	**M: 60.3 (5.4)**	94.4 (8.7)	**43.5 (4.1)**	89.4 (5.2)	**51.6 (4.4)**
	F: 107.8 (8.4)	**F: 50.5 (5.1)**	F: 80.4 (8.0)	**F: 61.9 (8.1)**

The effects of prenatal alcohol exposure (PAE) on basal and ethanol-evoked neuroimmune gene expression were assessed in either adolescence or adulthood. Control (free-choice liquid diet; FCL) and PAE-exposed pups matured until adolescence (P35) or adulthood (P90), at which time they were challenged with an acute gavage of either water (VEH) or ethanol (EtOH) (4 g/kg). Three hours after drug challenge, brains were collected for gene expression assessment in the hippocampus, amygdala, and paraventricular nucleus of the hypothalamus (PVN). Values shown are means for each group, with standard error of the mean (SEM) shown in parentheses. Gene abbreviations: ^a^interleukin-1 beta; ^b^tumor necrosis factor alpha; ^c^chemokine (C-X3-C motif) ligand 1 (also known as fractalkine); ^d^chemokine (C-X3-C motif) ligand 1 receptor (also known as fractalkine receptor). In these analyses, there were no instances in which a 3-way interaction between Sex, PAE, and Ethanol Challenge were observed. Since the majority of significant effects involved main effects of Ethanol Challenge or PAE, this table highlights these differences by indicating main effects of EtOH challenge with bold-faced text, and significant main effects of PAE with italics. However, there were a few analyses in which a significant 2-way interaction involving Sex and either PAE or Ethanol Challenge were observed. In order to show these Sex differences, means for males and females are presented separately in the table. In doing so, the inclusion of eight group means make highlighting differences from post hoc analysis of these 2-way interactions confusing to interpret in table format. Thus, additional formatting is not included for interactions involving Sex beyond group means.

### Ethanol Challenge-Induced Alterations in Cytokine Expression

**Figure 3:**
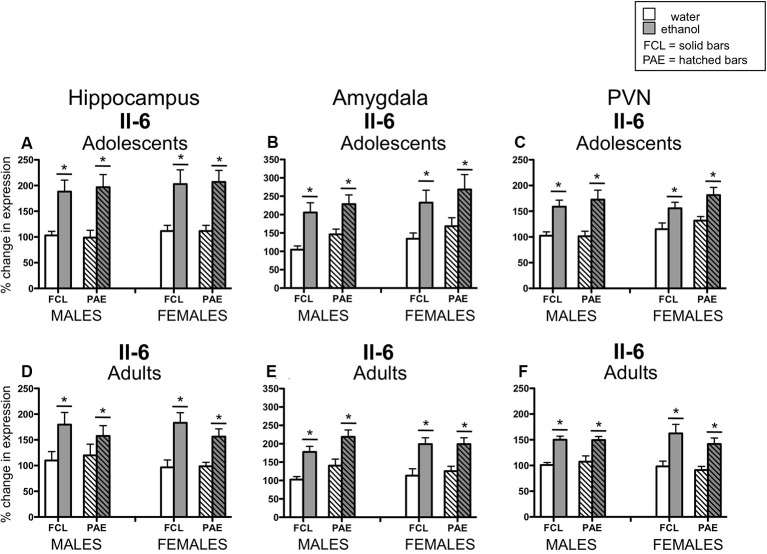
Adolescent (top row) and adult (bottom row) male and female rats were given an acute intragastric (i.g.) challenge of tap water (white bars) or 4-g/kg ethanol (EtOH; 20% v/v; gray bars), with brains collected for analysis 3 h after intubation. Half of the animals were offspring from mothers exposed to a prenatal alcohol diet (PAE groups; hatched bars), whereas the other half of the animals were born to dams that experienced a free-choice liquid diet (FCL groups; solid bars). *Interleukin (Il)-6* gene expression was examined in the hippocampus (panels **A,D**), amygdala (panels **B,E**) and paraventricular nucleus of the hypothalamus (PVN; panels **C,F**), with data calculated as a relative change in gene expression using the 2^−ΔΔC(t)^ method. Glyceraldehyde 3-phosphate dehydrogenase (GAPDH) was used as a reference gene and adult, male, FCL, water-exposed rats were used as the ultimate control group. Bars denote group means ± standard error of the mean (represented by vertical error bars). Data for adolescents and adults were analyzed separately, with the main effects of Drug Challenge signified by a horizontal line with an asterisk (*) above.

**Figure 4:**
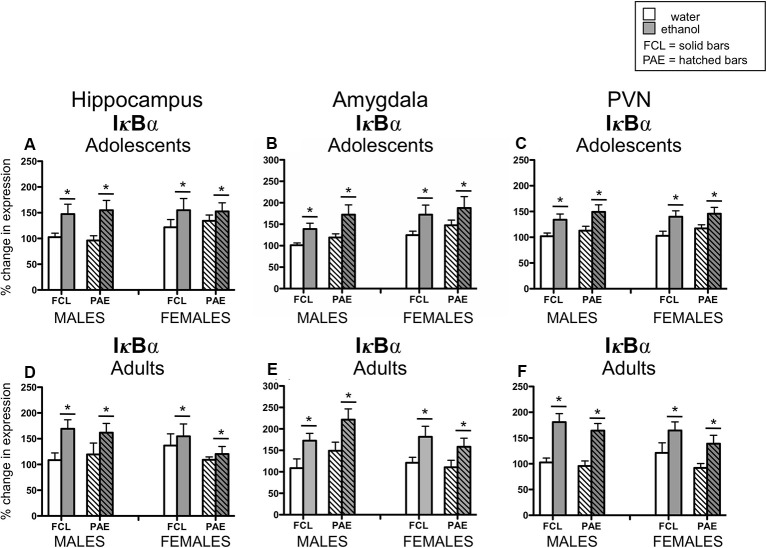
Adolescent (top row) and adult (bottom row) male and female rats were given an acute intragastric (i.g.) challenge of tap water (white bars) or 4-g/kg ethanol (EtOH; 20% v/v; gray bars), with brains collected for analysis 3 h after intubation. Hatched bars represent offspring born to mothers exposed to a prenatal alcohol diet (PAE groups), whereas solid bars denote offspring from dams that experienced a free-choice liquid diet (FCL groups). Expression of *IκBα* was examined in the hippocampus (panels **A,D**), amygdala (panels **B,E**) and paraventricular nucleus of the hypothalamus (PVN; panels **C,F**), with data calculated as a relative change in gene expression using the 2^−ΔΔC(t)^ method. Glyceraldehyde 3-phosphate dehydrogenase (GAPDH) was used as a reference gene and adult male FCL offspring challenge with water used as the ultimate control group. Bars denote group means ± standard error of the mean (represented by vertical error bars). Data for adolescents and adults were analyzed separately, with the main effects of Drug Challenge indicated by a horizontal line with an asterisk (*) above.

Acute challenge with a binge-like dose of ethanol resulted in significant increases in expression of *Il-6* (see Figure 3) in all three brain regions examined and at both ages (main effect of Drug Challenge at PD35 for *Il-6* in hippocampus: *F*_(1,70)_ = 42.21, *p* ≤ 0.000001, amygdala: *F*_(1,70)_ = 26.71, *p* ≤ 0.00001, and PVN: *F*_(1,70)_ = 35.76, *p* ≤ 0.000001; main effect of Drug Challenge at PD90 for *Il-6* in hippocampus: *F*_(1,61)_ = 23.95, *p* ≤ 0.00001; amygdala: *F*_(1,62)_ = 43.98, *p* ≤ 0.00001; and PVN: *F*_(1,65)_ = 49.33, *p* ≤ 0.000001). Similarly, an ethanol-related increase in expression of *IκBα* (see Figure 4) was also observed at PD35 and at PD90 in all brain areas analyzed [main effect of Drug Challenge at PD35 for *IκBα* in hippocampus: *F*_(1,70)_ = 11.49, *p* ≤ 0.001, amygdala: *F*_(1,70)_ = 13.33, *p* ≤ 0.001, and PVN: *F*_(1,70)_ = 20.11, *p* ≤ 0.0001; main effect of Drug Challenge at PD90 for *IκBα* in hippocampus: *F*_(1,61)_ = 6.62, *p* ≤ 0.05; amygdala: *F*_(1,54)_ = 18.35, *p* ≤ 0.00001; and PVN: *F*_(1,64)_ = 31.89, *p* ≤ 0.000001]. Although the stimulatory effect of ethanol challenge on hippocampal *IκBα* expression seemed less robust among the female offspring (especially the PAE females), Sex did not significantly interact with this ethanol effect. Furthermore, in none of these instances did the effects of ethanol challenge interact with Sex or Prenatal exposure to ethanol.

In contrast to the ethanol-related increases in gene expression noted above, in some cases, acute ethanol challenge resulted in significant reductions in neuroinflammatory gene expression. For example, expression levels of *Il-1β* (Table 2) were significantly suppressed among both adolescents and adults during acute intoxication in the hippocampus (main effect of Drug Challenge for adolescents: *F*_(1,70)_ = 9.99, *p* ≤ 0.01; for adults: *F*_(1,61)_ = 32.80, *p* ≤ 0.00001), amygdala (for adolescents: *F*_(1,70)_ = 19.98, *p* ≤ 0.0001; for adults: *F*_(1,62)_ = 10.83, *p* ≤ 0.001), and PVN (for adolescents: *F*_(1,70)_ = 22.51, *p* ≤ 0.0001; for adults: *F*_(1,65)_ = 8.18, *p* ≤ 0.01). The analysis of *Tnfα* expression levels also revealed an ethanol-associated decrease in all 3 brain areas examined (Table 2), with both adolescents (main effect of Drug Challenge in the hippocampus *F*_(1,70)_ = 25.12, *p* ≤ 0.00001; amygdala *F*_(1,70)_ = 49.78, *p* ≤ 0.00001; and PVN *F*_(1,70)_ = 58.95, *p* ≤ 0.000001) and adults (main effect of Drug Challenge in the hippocampus *F*_(1,61)_ = 59.61, *p* ≤ 0.000001; amygdala *F*_(1,62)_ = 97.04, *p* ≤ 0.00001; and PVN *F*_(1,65)_ = 55.93, *p* ≤ 0.000001) exhibiting this suppression following ethanol challenge. When expression levels of *Fractalkine* (*CX_3_CL-1*) and *Fractalkine receptor* (*CX_3_CL-1R*) were examined in the hippocampus and amygdala (Table 2), again, acute ethanol intoxication generally showed patterns of decreased gene expression that were small in magnitude but statistically significant. For adolescents, acute ethanol slightly but significantly suppressed *CX_3_CL-1R* expression, but only in the hippocampus (main effect of Drug Challenge: *F*_(1,70)_ = 9.94, *p* ≤ 0.01). Adults likewise demonstrated an ethanol-induced reduction in *CX_3_CL-1R* expression in hippocampus (*F*_(1,63)_ = 15.87, *p* ≤ 0.001) and amygdala (*F*_(1,62)_ = 9.12, *p* ≤ 0.01). Additionally, adults exhibited significant reductions in *CX_3_CL-1* levels in the hippocampus (*F*_(1,63)_ = 5.92, *p* ≤ 0.05), with *post hoc* analysis revealing that only adult females demonstrated a significant reduction in this gene in the amygdala [Sex × Drug Challenge: *F*_(1,62)_ = 4.32, *p* ≤ 0.05].

### Effects of Prenatal Alcohol Exposure on Neuroimmune Gene Expression

Although this model of prenatal ethanol exposure did not significantly interact with the stimulatory effects of ethanol challenge on *Il-6* and *IκBα* expression, ethanol-induced suppression of *Tnfα* was impacted by PAE in the PVN for adolescents (PAE × Drug Challenge interaction: *F*_(1,70)_ = 5.76, *p* ≤ 0.05), as well as in the hippocampus for adults (PAE × Drug Challenge interaction: *F*_(1,61)_ = 7.77, *p* ≤ 0.01; see Table 2). *Post hoc* analysis demonstrated that, among adolescents, PAE reduced *Tnfα* levels in water-challenged controls, which led to a less marked reduction in expression of *Tnfα* when this group was compared to the PAE ethanol-challenged rats. A comparable pattern of changes was observed for *Tnfα* expression in the hippocampus for adults. Thus, in the limited cases in which PAE affected ethanol alterations in neuroinflammatory markers, it blunted ethanol-related suppression of gene expression.

Overall, PAE effects were more often statistically observed in adults compared to adolescents, and the magnitude of the PAE effects was less marked than those induced by ethanol challenge. A main effect of PAE was revealed for adults when *Il-1β* expression in the PVN was analyzed (Table 2; *F*_(1, 65)_ = 4.19, *p* ≤ 0.05), and also for adolescents when *CX_3_CL-1R* expression in the hippocampus was examined (Table 2; *F*_(1,70)_= 9.94, *p* ≤ 0.05). Moreover, these PAE effects often interacted with Sex. For example, in adults, there was a significant Sex × PAE interaction in the hippocampus for *Tnfα* (*F*_(1,61)_ = 10.86, *p* ≤ 0.01), *CX_3_CL-1* (*F*_(1,63)_ = 5.06, *p* ≤ 0.05) and *CX_3_CL-1R* (*F*_(1,63)_ = 4.26, *p* ≤ 0.05) expression (Table 2). For all three of these gene targets, the Sex x PAE interaction demonstrated that, among males, prenatal alcohol exposure augmented expression when compared to the FCL controls. In contrast, PAE suppressed expression of these genes for female PAE offspring compared to FCL offspring. The same significant pattern was observed in the Sex x PAE interaction for *IκBα* expression in the amygdala in adults (*F*_(1,54)_ = 4.68, *p* ≤ 0.05) and for *Il-1β* expression in the PVN for adolescents (*F*_(1,70)_ = 4.57, *p* ≤ 0.05).

### Plasma Blood Ethanol and Corticosterone Concentrations

**Figure 5:**
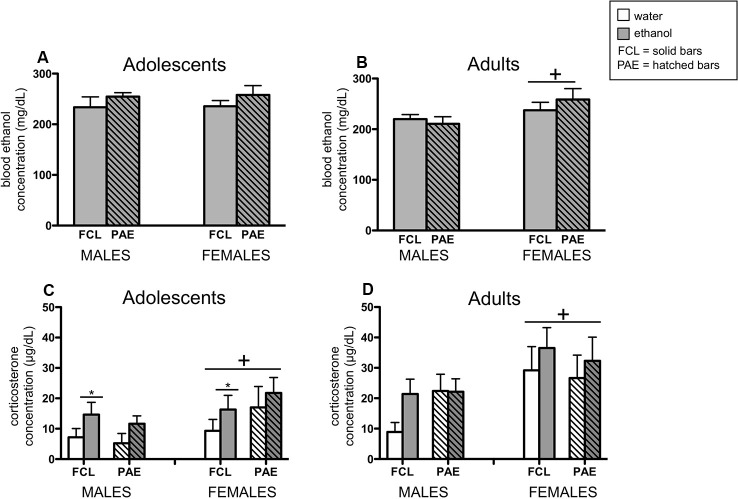
Plasma concentrations of ethanol (panels **A,B**) and corticosterone (panels **C,D**) were examined 3 h after an acute ethanol (4 g/kg; gray bars) or tap water (white bars) gastric gavage. Adolescents (left panels) and adults (right panels) were male and female offspring from mothers that experienced either a liquid diet containing ethanol (PAE groups; hatched bars) or a control liquid diet (FCL groups; solid bars) during gestational days 11–20. Bars denote group means ± standard error of the mean (represented by vertical error bars). Data for adolescents and adults were analyzed separately, and only ethanol-challenged rats included in the analyses of blood ethanol concentration. A plus symbol (+) indicates a significant main effect of Sex within a particular age group, whereas the line with an asterisk (*) denotes a significant main effect of the Drug Challenge.

In the analyses of BECs, only ethanol-exposed rats were included. However, plasma samples collected from vehicle-challenged rats were still measured in the ANALOX and these readings confirmed no measurable amount of ethanol in their blood (i.e., all vehicle controls exhibited values at the floor of the assay’s sensitivity; data not shown). Whereas adolescent rats challenged with ethanol had similar BECs regardless of their sex or prenatal condition (Figure 5A), adult female rats exhibited slightly but significantly higher BECs than their male counterparts after ethanol exposure (Figure 5B; main effect of Sex for adults: *F*_(1,35)_ = 4.15, *p* ≤ 0.05).

When plasma corticosterone concentrations were assessed (Figures 5C,D), a significant sex difference was observed for both adolescents and adults, with females exhibiting generally higher levels of corticosterone than males (main effect of Sex for adolescents: *F*_(1,71)_ = 4.34, *p* ≤ 0.05; for adults: *F*_(1,67)_ = 7.77, *p* ≤ 0.000001). Furthermore, adolescents challenged with ethanol had a significantly greater corticosterone response at this time point compared to rats that received the vehicle intubation (main effect of Drug Challenge for adolescents: *F*_(1,71)_ = 4.30, *p* ≤ 0.05).

## Discussion

Prenatal alcohol exposure leads to deficits in behavior and alterations in brain structure and function that are comparable in both humans and preclinical models. Additionally, PAE has been shown to alter neuroimmune function in offspring across development, with immediate and lasting changes in immune processes potentially contributing to the neurobehavioral consequences of FASD. To explore possible effects of PAE on both basal and ethanol-evoked cytokine responses in the CNS, the current experiments examined expression levels of several neuroimmune factors in the hippocampus, amygdala, and PVN of male and female PAE offspring at two developmental periods: mid-adolescence and adulthood. While ethanol was a potent modulator of neuroimmune factors in all three brain regions, the effects of PAE were much less marked in comparison. Furthermore, in most cases, PAE did not significantly influence ethanol-induced changes in any of the age or sex groups examined when they were compared to offspring that did not have gestational exposure to alcohol.

In the present experiments, adolescent and adult rats were challenged with a binge-like dose of ethanol, and then the expression of several neuroimmune factors assessed during peak intoxication. Ethanol markedly and significantly changed expression levels of the cytokines examined, with the results of this acute ethanol challenge confirming what we have previously observed (Doremus-Fitzwater et al., 2015, 2018; Gano et al., 2016, 2017). More specifically, ethanol intoxication elevated expression levels of *Il-6* and I*κBα* in all three brain regions examined, yet attenuated the expression of *Tnfα* and *Il-1β* mRNA. We have termed this ethanol intoxication-induced pattern of cytokine changes “rapid alterations in neuroimmune gene expression” (RANGE; Gano et al., 2016, 2017), and we have observed such changes in multiple strains of rats (Gano et al., 2017), at multiple ages (Doremus-Fitzwater et al., 2015; Gano et al., 2017), and in both sexes (Gano et al., 2017). Whereas repeated binge administrations of ethanol have been reported to lead to a state of heightened neuroimmune activation that is responsible for neuroinflammatory brain damage and some of the behavioral and cognitive effects of ethanol (Crews et al., 2017; Alfonso-Loeches et al., 2011; Montesinos et al., 2016), our laboratory and others have shown that a different pattern of responses is observed during the first few intoxicating exposures to ethanol challenge (Doremus-Fitzwater et al., 2015, 2018; Gano et al., 2016, 2017; Terasaki and Schwarz, 2017). While we have not yet identified the mechanisms responsible for these acute intoxication-related alterations in cytokines, acute ethanol exposure likely represents a non-pathogenic challenge that induces a sterile inflammatory response via activation of TLR4s through danger-associated molecular patterns (DAMPs), such as heat-shock proteins (e.g., hsp72) or high-mobility group box 1 (HMGB-1; Whitman et al., 2013; Crews et al., 2017).

Regardless of the mechanisms leading to ethanol intoxication-associated changes in brain cytokines, PAE effects were less pronounced overall. Only a few significant instances of PAE effects were observed: in adults, PAE led to slight but significant reductions in *Il-1β* expression in the PVN in adults, and *CX_3_CL-1R* expression in the hippocampus of PAE adolescents. Furthermore, PAE did not substantially impact ethanol-evoked responses, with the limited cases in which PAE significantly affected ethanol alterations in neuroinflammatory markers demonstrating that PAE blunted ethanol-related suppression of gene expression. Although not significant, it is worth noting that there was also a subtle but consistent augmentation of ethanol-induced increases in *Il-6* and *IκBα* expression in male and female adolescents and male adults. Using the same exposure model as in the present study, we have previously demonstrated alterations in ethanol-induced chemosensory plasticity that are important fundamental contributors to postnatal avidity for ethanol. For example, using this model, young rats exposed to gestational ethanol show enhanced ethanol intake (Youngentob et al., 2007b), as well as behavioral responses to ethanol odor that were mediated, in part, by an effect of maternal ethanol treatment on the neural response of the olfactory epithelium (Youngentob et al., 2007a). Moreover, prior fetal exposure increased EtOH intake, in part, by decreasing the generally aversive flavor properties of ethanol’s quinine-like bitter taste, capsaicin-like oral burning sensation, and aversive odor attributes (Glendinning et al., 2012; Youngentob and Glendinning, 2009). More recently, we have shown that fetal alcohol-induced attenuation in orosensory behavioral responses to later ethanol exposure is mediated by a reduction in the responsiveness of taste nerves and trigeminal chemosensory neurons to ethanol and its flavor components (Glendinning et al., 2017). Given the multitude of PAE effects observed with this model, and prior studies using heavier, binge-like, models of prenatal alcohol, we predicted pronounced PAE effects in the present study, which ultimately were not observed. However, more recently, researchers have suggested that low-to-moderate levels of PAE may result in subtle alterations in steady-state neuroinflammation that may not be observed under basal conditions. Indeed, the present experiments reported relatively few changes in basal neuroimmune gene expression that persisted into adolescence and adulthood with this relatively moderate PAE exposure paradigm, similar to a recent report (Terasaki and Schwarz, 2017)). Instead, it has been suggested that a subsequent perturbation, such as an immune or ethanol challenge (Bodnar et al., 2016; Noor and Milligan, 2018; Noor et al., 2017; Sanchez et al., 2017; Terasaki and Schwarz, 2016, 2017), may be required to unmask effects of prior PAE. In particular, an ethanol challenge during a later critical ontogenetic period such as adolescence has been thought to result in a situation in which these subtle lingering PAE effects might become visible, thus leading to increased vulnerability to ethanol-induced effects that might contribute to increased ethanol acceptance and drinking behaviors. However, in the present experiments, the PAE offsprings’ neuroimmune response to the drug challenge was not as different from control offspring as expected. One possibility for this outcome is that the single acute ethanol challenge administered here was not optimal for revealing such prenatal-postnatal interactions based on the dose utilized (i.e., we may be observing a ceiling effect after a 4 g/kg administration) or time point examined (i.e., PAE may alter the kinetics of the immune response to acute challenge and we did not select an optimal time to capture PAE *vs*. FCL effects in response to the challenge). To fully reveal lingering PAE effects, repeated exposures to ethanol might be required to observe more significant consequences of PAE (e.g., similar to the two consecutive ethanol binges in Terasaki and Schwarz, 2017), or there may be other time points (e.g., withdrawal; Topper et al., 2015) at which PAE effects would be more evident. Furthermore, it is possible that other brain regions, such as the prefrontal cortex (Terasaki and Schwarz, 2017), might be more sensitive to lasting effects of PAE on cytokines and chemokines, and their responsiveness to acute ethanol.

When the effects of PAE were compared in males and females, there were several instances in which the direction of the influence of PAE on gene expression levels was opposite in males *vs*. females. For adults, the hippocampus was especially differentially affected by PAE across sex—*Tnfα, CX_3_CL-1* and *CX_3_CL-1R* expression was potentiated in PAE males compared to FCL males, whereas PAE females exhibited decreased expression of these genes relative to control offspring. Expression levels of *IκBα* in the amygdala in adults, and *Il-1β* levels in the PVN in adolescents revealed this same pattern. That females and males showed different responses to PAE-induced changes in neuroimmune factors was not unexpected. Under normal conditions, developmental differences in neuroimmune system function are present, including sex differences in microglial colonization and structure, and expression of neuroinflammatory factors (Schwarz and Bilbo, 2012; Schwarz et al., 2012). Additionally, other laboratories have reported male *vs*. female differences in the effects of PAE on both basal and challenge-evoked alterations in cytokines and chemokines (Terasaki and Schwarz, 2016, 2017; Topper et al., 2015). These reports have been somewhat equivocal, however, with females sometimes exhibiting augmented responses and in other instances females demonstrating blunted responses. Likely, differences in PAE duration and dosing, as well as the timing of assessment, brain region of interest, and type of developmental challenge are all variables contributing to contrasting sex differences across studies.

When considering the effects of ethanol on the neuroimmune system, adolescence has emerged as a critical period that may be uniquely sensitive to both acute and long-term ethanol exposure (Crews and Vetreno, 2014). In our laboratory, we previously reported that, when age differences were present, adolescents exhibited blunted neuroimmune responses to either acute ethanol or immune challenge with LPS (Doremus-Fitzwater et al., 2015). A similar reduced response among adolescents to acute ethanol was also reported by Kane et al. (2014). Here, we examined both adolescents and adults with a history of PAE and then assessed their responses to acute binge-like ethanol on brain cytokines. Given the blunted response to acute ethanol in adolescents vs. adults without PAE history, we further anticipated that PAE would engender an even larger blunting to acute ethanol effects on neuroimmune factors among PAE adolescents. While our experimental design did not allow us to directly compare adolescents and adults (as they were two separate cohorts that were analyzed separately), when generally comparing regions and targets examined between adolescents and adults, adolescents mounted immune changes similar in pattern and in magnitude to those exhibited by adults. Thus, these data do not suggest a strong age difference in this RANGE response, as we would have expected. At this time, it is not clear why adolescents in this experiment mounted an ethanol-induced immune response similar to adults. It is possible that this discrepancy may be due to strain differences in developmental maturity of the neuroimmune system. The present experiments were conducted with Long Evans rats, whereas previous work was done in Sprague–Dawley rats at a slightly earlier age (Doremus-Fitzwater et al., 2015). More work needs to be done to clearly understand the sensitivity of adolescents to PAE and acute ethanol, with particular attention paid to variables such as these.

## Summary and Conclusions

Overall, the present findings indicate that moderate exposure to gestational alcohol leads to more subtle long-lasting effects on the neuroinflammatory milieu of the offspring. Furthermore, these results would suggest that, for most individuals and at most ages, the primary determinant of the neuroimmune response evoked by a binge-like ethanol challenge is the binge-like challenge, itself. Certainly, additional studies will be required to more fully address this issue. Children with fetal alcohol exposure may not always exhibit obvious or apparent consequences of such exposure, particularly if the prenatal exposure was modest or low in magnitude. However, the evidence is growing to suggest that low to moderate fetal alcohol exposure may cause insidious neuroimmune consequences that are only unmasked when future life events that trigger immune system responses are encountered. Thus, studies such as these are contributing to a growing understanding of how multiple developmental “hits” may enhance or suppress immune system function across ontogeny.

## Data Availability Statement

The datasets generated for this study are available on request to the corresponding author.

## Ethics Statement

The animal study was reviewed and approved by the Committee on Humane Use of Animals (CHUA) at SUNY-Upstate Medical University (previous employer for SY), and studies were conducted in accordance with the Public Health Service (PHS) policy on the Humane Care and Use of Laboratory Animals.

## Author Contributions

SY and LY prenatally exposed rats to alcohol in SY’s research laboratory when previously employed at Upstate Medical University (UMU), and also challenged offspring during adolescence or adulthood with ethanol or vehicle. TD-F, AG, and TD all participated in tissue sample collection at UMU, with TD-F, AG, and AV working to process brain tissue and blood samples in TD’s laboratory. TD-F, AV, and TD worked together to analyze data presented in the current manuscript, with TD-F and TD as the primary authors. All authors read, reviewed, and contributed substantially to the manuscript presented here.

## Conflict of Interest

The authors declare that the research was conducted in the absence of any commercial or financial relationships that could be construed as a potential conflict of interest.

## Footnotes

^1^statistics retrieved from: https://www.cdc.gov/ncbddd/fasd/data.html#ref
